# Machine learning algorithms to predict major bleeding after isolated coronary artery bypass grafting

**DOI:** 10.3389/fcvm.2022.881881

**Published:** 2022-07-28

**Authors:** Yuchen Gao, Xiaojie Liu, Lijuan Wang, Sudena Wang, Yang Yu, Yao Ding, Jingcan Wang, Hushan Ao

**Affiliations:** ^1^Department of Anesthesiology, Fuwai Hospital, State Key Laboratory of Cardiovascular Disease, National Center of Cardiovascular Diseases, Chinese Academy of Medical Sciences and Peking Union Medical College, Beijing, China; ^2^Department of Anesthesiology, The Affiliated Hospital of Qingdao University, Qingdao, China

**Keywords:** major bleeding, machine learning, prediction model, cardiac surgery, coronary artery bypass graft (CABG) surgery

## Abstract

**Objectives:**

Postoperative major bleeding is a common problem in patients undergoing cardiac surgery and is associated with poor outcomes. We evaluated the performance of machine learning (ML) methods to predict postoperative major bleeding.

**Methods:**

A total of 1,045 patients who underwent isolated coronary artery bypass graft surgery (CABG) were enrolled. Their datasets were assigned randomly to training (70%) or a testing set (30%). The primary outcome was major bleeding defined as the universal definition of perioperative bleeding (UDPB) classes 3–4. We constructed a reference logistic regression (LR) model using known predictors. We also developed several modern ML algorithms. In the test set, we compared the area under the receiver operating characteristic curves (AUCs) of these ML algorithms with the reference LR model results, and the TRUST and WILL-BLEED risk score. Calibration analysis was undertaken using the calibration belt method.

**Results:**

The prevalence of postoperative major bleeding was 7.1% (74/1,045). For major bleeds, the conditional inference random forest (CIRF) model showed the highest AUC [0.831 (0.732–0.930)], and the stochastic gradient boosting (SGBT) and random forest models demonstrated the next best results [0.820 (0.742–0.899) and 0.810 (0.719–0.902)]. The AUCs of all ML models were higher than [0.629 (0.517–0.641) and 0.557 (0.449–0.665)], as achieved by TRUST and WILL-BLEED, respectively.

**Conclusion:**

ML methods successfully predicted major bleeding after cardiac surgery, with greater performance compared with previous scoring models. Modern ML models may enhance the identification of high-risk major bleeding subpopulations.

## Introduction

Postoperative bleeding is a significant cause of morbidity and mortality in patients undergoing cardiac surgery, particularly after coronary artery bypass grafting (CABG) because they often are treated with potent antithrombotic drugs (1). Excessive bleeding after cardiac surgery is related to significant blood product transfusion, infections, longer mechanical ventilation, prolonged intensive care unit (ICU) stay, emergency surgical re-exploration, acute kidney injury, sepsis, and thromboembolic events (2–4). In addition, several studies have reported that major bleeding after cardiac surgery was also associated with increased hospital costs (5, 6). Therefore, identifying patients at high risk of bleeding will allow clinicians to take additional precautionary action to mitigate risk.

Several risk stratification models have been developed to support clinical decision, making such as the TRUST and WILL-BLEED risk scores (7, 8). All the models in current use are based on logistic regression (LR), which relied on manually inputting variables. Missing the complex interactions among features may result in model misspecification (9). Artificial intelligence is a rapidly growing field in cardiovascular medicine (10). Machine learning (ML) is a sub-field of artificial intelligence that may produce better predictive ability than traditional statistical analysis to predict postoperative outcomes. These approaches better account for high-order, non-linear interactions between predictors and can gain more stable predictions (11). Despite the success of ML in other clinical applications, no study has yet examined the utility of modern ML models to predict postoperative major bleeding in patients with isolated CABG.

The goal of this study was to develop ML models to accurately predict major bleeding after isolated CABG. We also compared the performance of several ML approaches with the reference LR model, the and the TRUST and WILL-BLEED scores.

## Materials and methods

### Data source and study population

We analyzed electronic health record data from Fuwai Hospital, Beijing, China from 1,045 consecutive adult patients who underwent elective isolated CABG between September 2017 and May 2018. The subjects were assigned randomly to training and test sets at a ratio of 7:3. This retrospective cohort study was approved by the Institutional Review Board with waived written consent.

### Study variables

We collected datasets that included demographics, surgery-related data, and clinical and laboratory parameters. The continuous anti-platelet therapy was provided only in patients with acute coronary syndrome presenting for CABG. All data were obtained from the electronic health records as in recent articles (12, 13). Our database initially comprised over 100 easily obtained variables. Features that were missing for more than 50% of cases were excluded. The final set of input features included 60 clinical variables. Missing values (Supplementary Table 2) were imputed using the mean imputation method, which replaces the missing values with the mean of the available cases.

### Definition of major bleeding

Development of postoperative major bleeding was defined according to the universal definition of perioperative bleeding (UDPB) in classes 3 and 4 (14, 15). The UDPB defines 5 perioperative bleeding classes, which are intended to characterize the severity of bleeding, regardless of its source. The details of the UDPB are presented in Supplementary Table 1.

### Statistical analysis

Data are presented as means with SD, medians and interquartile ranges (IQRs), or frequencies and proportions depending on variable type and distribution. Differences between groups were compared using the Student’s *t*-test for normally distributed continuous variables, the Mann-Whitney *U*-test for non-normally distributed continuous variables, and the Pearson’s chi-square test of independence for categorical variables.

To implement the ML algorithms, we randomly assigned patient data into a training set (70%) for model derivation and a testing set (30%) to be used for model validation. Assignment to the training/testing sets was stratified on primary outcome status.

To select features for LR models, we conducted a backward stepwise selection procedure based on the Akaike information criterion (AIC). Model development included several common ML methods, such as support vector machines (SVM), stochastic gradient boosting (SGBT), extreme gradient boosting (XGBoost), random forest (RF), conditional inference random forest (CIRF), boosted classification trees, Naïve Bayes (NB), and bagged classification and regression tree (CART). We report the variables included in each model in Supplementary Table 4. The details of the R package were presented in Supplementary Material. We conducted a fivefold cross-validation to tune the parameters of the ML models on the training set and determined the best hyperparameter. Discrimination was evaluated with the receiver-operating-characteristic curve (ROC) and the area under the ROC curve (AUROC) (16). To evaluate the performance of ML algorithms, we compared the ROC between the models using Delong’s test by the *pROC* package (17). Calibration, which is the agreement between predicted probabilities and observed frequencies of postoperative major bleeding, was assessed with calibration belts (18). The net reclassification improvement was used to quantify whether a new model provides clinically relevant improvements in prediction (19, 20). We graphically demonstrated the net benefit of each model through a range of threshold probabilities of the outcome as a decision curve. The variable importance from ensemble algorithms was also determined, with the top 20 important variables from each algorithm identified. Analysis code is available upon request. All statistical analyses were performed using R software (version 4.1.1; The Comprehensive R Archive Network).

## Results

### Baseline characteristics

We assigned 732 and 313 patients to the training and testing sets, respectively. The variables remained constant between the two sets (Supplementary Table 3). The postoperative major bleeding rate were similar between the training and test sets. Overall, the median age was 62 years (IQR 55–66 years) and 78.4% were men. At baseline, patients who developed a major bleeding event were older, had lower body surface area (BSA), and were more likely to have a history of anemia. Other demographics and perioperative variables of the training set are listed in Table 1.

**TABLE 1:** Baseline characteristics of the training set.

Variables	Total (*n* = 732)	No major bleeding (*n* = 680)	Major bleeding (*n* = 52)	*P*
Age (years)	62 (55–67)	61 (55–66)	65 (59–69)	0.017
Male, n (%)	559 (76.37)	524 (77.06)	35 (67.31)	0.127
Height (m)	1.68 (1.62–1.72)	1.69 (1.63–1.72)	165 (1.60–1.70)	0.007
Weight (kg)	72.00 (65.00–80.00)	72.00 (65.00–80.00)	67.25 (62.00–74.25)	0.001
BSA (m^2^)	1.83 ± 0.17	1.84 ± 0.17	1.76 ± 0.2	0.002
Smoking history, n (%)	375 (51.23)	349 (51.32)	26 (50.00)	0.886
Angina, n (%)	699 (95.49)	647 (95.15)	52 (100.00)	0.160
Myocardial infarction, n (%)	62 (8.47)	57 (8.38)	5 (9.62)	0.961
Arrhythmia, n (%)	22 (3.01)	20 (2.94)	2 (3.85)	0.665
Previous surgery, n (%)	176 (24.04)	164 (24.12)	12 (23.08)	0.866
Diabetes, n (%)	272 (37.16)	260 (38.24)	12 (23.08)	0.036
Hyperlipidemia, n (%)	603 (82.38)	559 (82.21)	44 (84.62)	0.850
Hypertension, n (%)	459 (62.70)	434 (63.82)	25 (48.08)	0.026
Kidney failure, n (%)	21 (2.87)	20 (2.94)	1 (1.92)	> 0.99
Dialysis, n (%)	17 (2.32)	17 (2.50)	0 (0)	0.625
Chronic pulmonary disease, n (%)	11 (1.50)	11 (1.62)	0 (0)	> 0.99
Congestive heart failure, n (%)	10 (1.37)	8 (1.18)	2 (3.85)	0.155
Anemia, n (%)	183 (25.00)	162 (23.82)	21 (40.38)	0.012
Peripheral vascular disease, n (%)	174 (23.77)	161 (23.68)	13 (25.00)	0.866
Venous disease, n (%)	46 (6.28)	43 (6.32)	3 (5.77)	> 0.99
Cerebrovascular disease, n (%)	84 (11.48)	77 (11.32)	7 (13.46)	0.651
Previous PTCA, n (%)	16 (2.19)	14 (2.06)	2 (3.85)	0.316
Previous thrombolysis, n (%)	5 (0.68)	4 (0.59)	1 (1.92)	0.309
CHD family history, n (%)	77 (10.52)	69 (10.15)	8 (15.38)	0.240
Preoperative statin use, n (%)	307 (41.94)	286 (42.06)	21 (40.38)	0.885
Preoperative anticoagulant use, n (%)	639 (87.30)	596 (87.65)	43 (82.69)	0.284
Antiplatelet drugs pause < 5 days, n (%)	12 (1.64)	10 (1.47)	2 (3.85)	0.207
Left main coronary artery disease, n (%)	134 (18.31)	127 (18.68)	7 (13.46)	0.457
RBC (×10^12^/L)	4.44 ± 0.52	4.45 ± 0.51	4.32 ± 0.54	0.064
WBC (×10^9^/L)	6.36 (5.31–7.41)	6.36 (5.30–7.41)	6.31 (5.35–7.41)	0.934
PLT (×10^9^/L)	208 (176–247)	209 (177–249)	199 (167–228)	0.066
Platelet distribution width (fL)	12.30 (11.20–13.80)	12.30 (11.20–13.80)	12.35 (11.20–13.90)	0.841
Platelet volume (fL)	10.50 (10.00–11.20)	10.5 (10.00–11.20)	10.45 (10.07–11.33)	0.637
Platelet-large cell ration (%)	29.40 (24.70–34.90)	29.40 (24.58–34.73)	28.65 (26.15–35.42)	0.653
Thrombocytocrit (%)	0.22 (0.19–0.26)	0.22 (0.19–0.26)	0.21 (0.19–0.23)	0.096
Hemoglobin (g/L)	137 (126–146)	137 (127–146)	137 (120–143)	0.045
Total protein (g/L)	66.05 (62.90–70.10)	66.15 (63.00–70.30)	65.20 (61.65–68.03)	0.061
Albumin (g/L)	40.80 (38.70–43.42)	40.85 (38.70–43.60)	40.40 (38.48–42.20)	0.097
Potassium (mmol/L)	4.03 (3.77–4.25)	4.03 (3.77–4.26)	4.02 (3.72–4.13)	0.326
Sodium (mmol/L)	141.10 (139.12–143.01)	141.17 (139.13–143.01)	140.66 (138.74–142.74)	0.630
Calcium (mmol/L)	2.26 (2.19–2.34)	2.26 (2.19–2.34)	2.27 (2.19–2.33)	0.887
Glucose (mmol/L)	5.34 (4.69–6.54)	5.36 (4.69–6.55)	5.05 (4.64–6.18)	0.167
BUN (mmol/L)	5.23 (4.19–6.44)	5.29 (4.19–6.44)	5.07 (4.22–6.30)	0.431
Creatine (μmol/L)	82.00 (70.48–94.00)	82.00 (70.27–94.00)	84.29 (72.30–95.43)	0.527
GFR (ml/min/1.73 m^2^)	84.29 (71.51–93.94)	84.52 (72.14–94.24)	78.37 (64.62–92.18)	0.051
HSCRP (mg/L)	1.38 (0.66–3.10)	1.42 (0.67–3.10)	1.08 (0.50–2.99)	0.293
NT-proBNP (pg/ml)	156.35 (62.98–369.52)	154.05 (62.27–373.00)	177.75 (86.90–315.28)	0.532
PT (s)	13.10 (12.60–13.60)	13.10 (12.60–13.53)	13.30 (12.97–13.62)	0.045
INR (R)	1.00 (0.96–1.04)	1.00 (0.95–1.04)	1.02 (0.99–1.06)	0.047
CPB or not, n (%)	477 (65.16)	444 (65.29)	33 (63.46)	0.765
Operation time (h)	3.80 (3.30–4.50)	3.80 (3.30–4.40)	3.90 (3.38–4.93)	0.275
Blood loss (ml)	589.54 (75.17)	587.76 (68.81)	612.69 (131.82)	0.021
Intraoperative transfusion, n (%)	14 (1.91)	7 (1.03)	7 (13.46)	< 0.001
Intraoperative urine output (ml/kg/h)	3.16 (2.02)	3.13 (2.01)	3.54 (2.11)	0.159
Hemoglobin decrease (g/L)	25.00 (16.00–33.00)	25.00 (16.00–33.00)	27.00 (14.25–33.00)	0.961
Postoperative first Creatine (μmol/L)	70.27 (60.88–81.90)	70.31 (60.88–81.80)	68.97 (60.85–84.61)	0.707
Postoperative first NT-proBNP (pg/ml)	602.10 (350.15–1060.75)	596.30 (342.08–1042.75)	690.90 (409.08–1124.25)	0.108
Preoperative hospital LOS (d)	6 (4–9)	6 (4–9)	6 (4–8)	0.596
TRUST score	2 (1–3)	2 (1–3)	2 (1–3)	0.002
WILL-BLEED score	1 (1–3)	1 (1–3)	3 (1–4)	0.013

BSA, body surface area; PTCA, percutaneous transluminal coronary angioplasty; CHD, coronary heart disease; RBC, red blood cell; WBC, white blood cell; PLT, platelet; BUN, blood urea nitrogen; GFR, glomerular filtration rate; HSCRP, high sensitivity C reactive protein; NT-proBNP, N-terminal prohormone of brain natriuretic peptide; PT, prothrombin time; INR, international normalized ratio; LOS, length of stay.

### Development of postoperative major bleeding prediction model

The overall major bleeding rate was 7.1% (74 of 1,045 patients) in this study population. The variables included in each model are presented in Supplementary Table 4. Multivariable LR analysis with backward stepwise variable selection based on AIC is shown in Table 2. ROC analysis showed an adequate discriminatory ability of the LR model [0.702 (0.577–0.827)]. The AUC of the CRIF and SVM models performed significantly better (*p* < 0.05) than the LR model in the test set. The discrimination ability of models selected in the testing set is presented in Table 3.

**TABLE 2:** Final logistic regression model.

Variables*	OR (95%CI)	*P*-value*
BSA	0.018 (0.002–0.133)	<0.001
Diabetes	0.537 (0.256–1.055)	0.083
Hypertension	0.426 (0.577–0.827)	0.008
Total protein	0.931 (0.882–0.981)	0.008
Creatine	1.023 (1.004–1.041)	0.014
NT-proBNP	1.000 (0.999–1.000)	0.025
Blood loss	1.004 (1.000–1.007)	0.029
Intraoperative transfusion	21.968 (5.879–84.392)	<0.001

OR, odds ratio; NT-proBNP, N-terminal prohormone of brain natriuretic peptide.

*The backward stepwise selection procedure base on Akaike information criterion was performed to select variables.

**TABLE 3:** Prediction models of postoperative major bleeding in the test set.

Models	AUC (95%CI)	*P*-value*	*P*-value^†^	*P*-value^‡^
TRUST	0.629 (0.517–0.741)			
WILL-BLEED	0.557 (0.449–0.665)			
Logistic regression	0.702 (0.577–0.827)	0.347	0.103	
Support vector machine	0.792 (0.678–0.907)	0.016	0.002	0.031
Xgboost	0.802 (0.691–0.913)	0.028	<0.001	0.120
Random forest	0.810 (0.719–0.902)	0.005	<0.001	0.084
Conditional inference random forest	0.831 (0.732–0.930)	0.002	<0.001	0.027
Stochastic gradient boosting	0.811 (0.739–0.883)	<0.001	<0.001	0.073
Naïve Bayes	0.687 (0.561–0.813)	0.468	0.059	0.842
Bagged CART	0.791 (0.706–0.876)	0.008	<0.001	0.098
Boosted classification trees	0.794 (0.675–0.913)	0.007	0.003	0.117

AUC, the area under the receiver operating characteristic curve; CART, classification and regression tree.

*Compared with TRUST.

^†^Compared with WILL-BLEED.

^‡^Compared with the logistic regression model.

**FIGURE 1:**
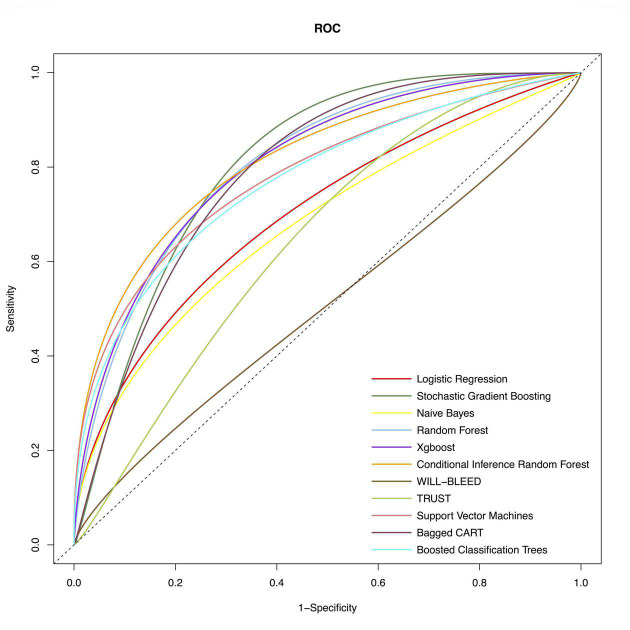
Receiver-operating-characteristics (ROC) curves of the conventional model and machine learning models for postoperative major bleeding in the test set. The corresponding values of the area under the receiver-operating-characteristics curve (AUC) for each model are presented in Table 2.

We used the following ML methods in putting all the variables, including SVM, SGBT, XGBoost, RF, CIRF, boosted classification trees, NB, and bagged CART to predict major bleeding after isolated CABG. The ROC of the evaluated models are presented in Figure 1. The AUC of the CIRF model was 0.831 (0.732–0.930), which was the highest among the ML models. The SGBT and RF models achieved the next and the third-highest AUC value of 0.820 (0.742-0.899) and 0.810 (0.719–0.902), respectively. The AUC values of TRUST and WILL-BLEED for the prediction of major bleeding were 629 (0.517–0.741) and 0.557 (0.449–0.665), respectively. All ML models except the NB model performed remarkably better than the TRUST and WILL-BLEED risk scores (*p* < 0.05).

**FIGURE 2:**
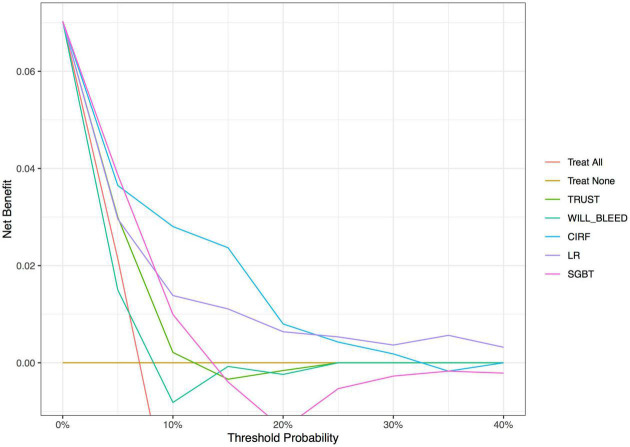
Decision curve analysis of the conventional models and machine learning-based models. The *X*-axis indicates the threshold probability and *Y*-axis indicates the net benefit. The red solid line indicates the net benefit of all patients developing postoperative major bleeding events. Ochre’s solid line indicates the net benefit of no patients developing postoperative major bleeding events. LR, logistic regression; CIRF, conditional inference random forest; SGBT, stochastic gradient boosting.

The decision curve analysis (Figure 2) showed that the net benefit of ML surpassed that of the conventional scoring systems throughout the range of threshold probabilities, indicating that the ML models yielded a higher net benefit. The threshold probability is a level of certainty, above which the patient or physician would choose to intervene.

**FIGURE 3:**
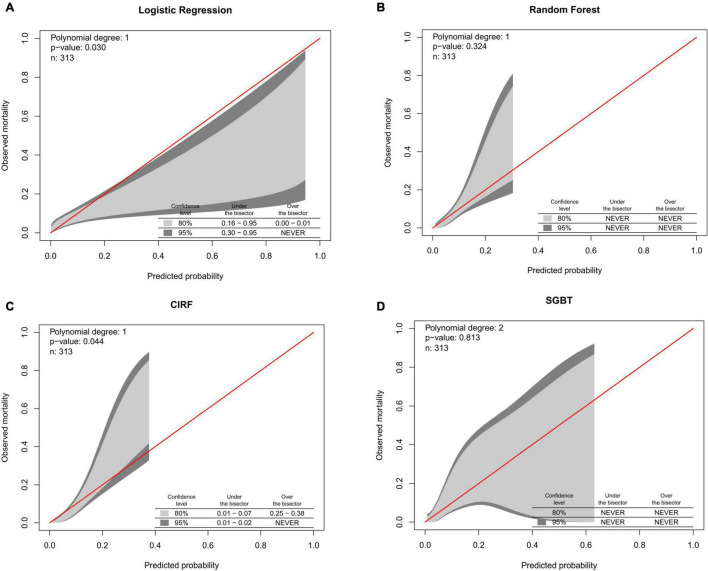
Calibration belts of **(A)** logistic regression. **(B)** Random forest. **(C)** Conditional inference random forest. **(D)** Stochastic gradient boosting for postoperative major bleeding prediction in the test set.

The calibration belts of the ML models and the LR model for major bleeding are shown in Figure 3. The RF and SGBT models showed better calibration, while CIRF and LR models were not well-calibrated.

### Variable importance of machine learning models

**FIGURE 4:**
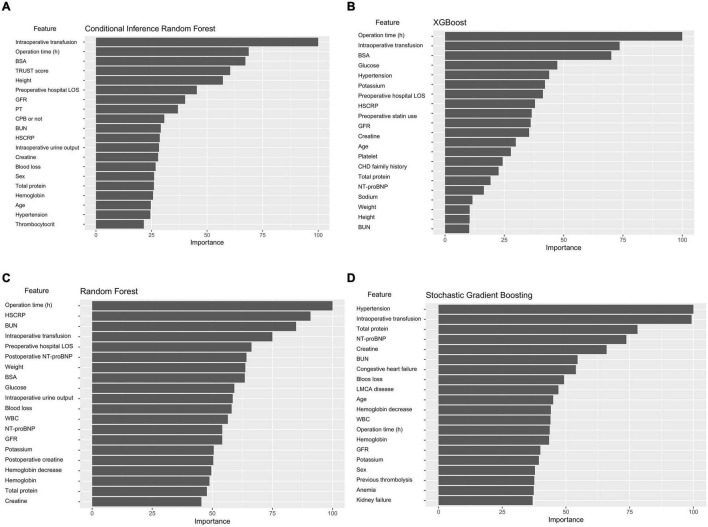
Variable importance of predictors in the ML models. The variable importance is a scaled measure to have a maximum value of 100. The predictors with variable importance of the top 20 are shown. **(A)** Conditional inference random forest. **(B)** Xgboost. **(C)** Random forest. **(D)** Stochastic gradient boosting.

Figure 4 shows the importance matrix plot of machine learning models. To gain insights into the relevance of each predictor, Figure 4A summarizes the 20 most important predictors used in the CIRF model. Of the top 20 most important features, 15 were preoperative variables and 5 were intraoperative variables. In the CIRF model, intraoperative transfusion was the most important variable in predicting postoperative major bleeding, followed by operation time and BSA. The determinative factors in the ML and logistic models were roughly the same.

## Discussion

The clinical application of ML has emerged as a new tool to improve patient care. In this study, we compared the predictive accuracy of models for major bleeding after isolated CABG among ML techniques, a traditional statistical approach, and previous risk scoring models. The models developed using ML algorithms better predicted major bleeding events than conventional scoring systems, such as TRUST and WILL-BLEED.

To date, accurate prediction models that use statistical models for the risk of postoperative major bleeding have been lacking. The TRUST and WILL-BLEED scores using clinical and laboratory variables were developed using only preoperative factors (7, 8). These two prediction models were derived from multicenter databases and have demonstrated accurate prediction of bleeding or transfusion in cardiac surgery patients. However, these approaches showed poor performance in our dataset. Our study demonstrated the value of intraoperative and immediate postoperative data, which reflected the surgical-related factors to bleeding prediction. Integrating these features may elucidate the improved discrimination of our LR model compared with the TRUST and WILL-BLEED scores.

The LR model used in this study shares several risk factors for bleeding common to other prediction models. Interestingly, patients with hypertension could have a lower risk of postoperative major bleeding in this study. Patients with hypertension undergoing CABG had greater blood pressure variability, and lability as a protective marker demonstrating that autonomic nervous system integrity is associated with improved outcomes (21, 22). A retrospective analysis found that hypertension is a positive prognostic indicator in most patients undergoing anesthesia and surgery (23). A prior study has demonstrated that patients who developed major bleeding have lower systolic blood pressures than those who did not have major bleeding (24). These may be potential explanations for this contrary perception.

By capturing the non-linear association of predictors ML approaches may improve prediction in major bleeding (25). ML models in this study performed the best but at the cost of possibly increased complexity and lessened clinical significance. While overfitting in conventional models is often problematic, this study adopted a cross-validation method to mitigate the overfitting of ML models. A recent systematic review on the application of ML methods for predicting operative mortality following cardiac surgery demonstrated that ML models provide better discrimination ability (26). The XGBoost ML algorithm offered modest improvements across a variety of model performance measures compared with The Society of Thoracic Surgeons Predicted Risk of Mortality (STS PROM) (27). Zea-Vera and associates (28) recently developed an ML model to predict mortality, major morbidity, high total hospitalization cost, and 30-day re-admission. Nevertheless, Benedetto et al. (29) reported that the performance of ML techniques was not superior to an LR model in predicting operative mortality after cardiac surgery. Therefore, the additional value of ML in the development of prediction models for a variety of clinical conditions needs further investigation.

Decision curve analysis was developed as a method to determine whether the use of a prediction model in the clinic to inform decision-making would do more good than harm (30). This analysis indicated that the CIRF model improved the net benefit for predicting the postoperative major bleeding over the LR model and TRUST and WILL-BLEED scores. The threshold ranges displayed above the curves (in Figure 2) indicate how the ML models will apply to clinical practice. For example, if a patient’s threshold probability is 15%, according to the decision curve, the use of the CIRF model adds more benefit than either the treat-all-patients scheme or the treat-none scheme and even the other models.

Preventing postoperative bleeding is imperative to improve patient outcomes and decrease hospital costs. Prompt identification of patients at high-risk could help allow deployment of additional resources to achieve better outcomes, including precautions, such as preoperative correction of anemia, earlier discontinuation of antiaggregant drugs, detailed coagulation tests, better surgical homeostasis, and various procoagulant agent during the operation (31). Understanding which patients are at high risk for postoperative bleeding will also help guide future practice and mitigate worse outcomes associated with bleeding. Meticulous surgical technique aimed to reduce surgical site bleeding seems to be essential to prevent severe bleeding, exposure to blood products, and improve the prognosis after CABG.

Several potential limitations exist in our study. First, because it employed retrospective data from a single center at a tertiary hospital, the generalizability of these models will need further validation. Second, the study identified the most important variables concerning predicting postoperative major bleeding events, but we could not obtain certain degrees of risk, such as the relative risk, which is a common limitation of ML algorithms. In addition, we were only able to provide a comparison with the TRUST and WILL-BLEED scores and were unable to compute other risk scores because we lacked appropriate data. Finally, the most important variables reported in the ML model are not modifiable and accurate risk prediction may not be followed by improved patient outcomes. Thus, further prospective studies are required to evaluate the application of ML-based predictive models to clinical practice for the reduction of postoperative major bleeding risks.

## Conclusion

Our study demonstrated that the ML technique of CIRF showed significantly better performance than the traditional LR analysis or previous scoring models in predicting postoperative major bleeding events for isolated CABG. By capturing the non-linear association of predictors, the ML approaches may facilitate optimal candidate selection and prognostication of patients undergoing isolated CABG. Future prospective studies are required to evaluate the application of predictive models based on ML to clinical practice and reduce bleeding risks.

## Data availability statement

The dataset generated and analyzed during the current study is available from the corresponding author upon reasonable request.

## Ethics statement

The studies involving human participants were reviewed and approved by the Ethics Committee of Fuwai Hospital. Written informed consent for participation was not required for this study in accordance with the national legislation and the institutional requirements.

## Author contributions

YG and HA: designed the study, collected and analyzed the data, drafted the manuscript, and submitted the final version. XL and HA: writing–reviewing and editing. YG, XL, and LW: visualization, coordinated, and supervised data collection. SW, YY, YD, and JW: collected, checked, and validated the data. All authors read and approved the manuscript for submission.
