# Supplementary material for: Machine learning algorithms to predict major bleeding after isolated coronary artery bypass grafting
Source: Front Cardiovasc Med. 2022 Jul 28;9:881881. doi: 10.3389/fcvm.2022.881881 (PMC9366116; doi:10.3389/fcvm.2022.881881)
Supplement: Supplementary file 1 [file Data_Sheet_1.pdf]

Machine learning algorithms to predict major bleeding after isolated coronary artery bypass grafting

Supplementary Material

Methods

5-fold cross validation

The performance of machine learning models was evaluated using a stratified 5-fold cross-validation procedure during hyperparameter tuning. In this procedure, the dataset is randomly divided into 5 equal folds, each with approximately the same number of events. Five validation experiments are then performed, with each fold used in rotation as the validation set, and the remaining 4 folds as the training set. Therefore, each data point is used once for testing and 4 times for training, resulting in 5 experimental machine learning models trained on 80% fractions. The average of the validation results from 5 experimental models is calculated to provide a measure of the overall performance.

Variable importance

For classification models, each predictor will have a separate variable importance for each class. All measures of importance are scaled to have a maximum value of 100.

Random forest / Conditional inference random forest

To examine the importance of each predictor in the random forest models, we used permutation-based variable importance that is determined by the normalized average value of difference between prediction accuracy of the out-of-bag estimation and that of the same measure after permutating each predictor.

Xgboost / Stochastic gradient boosting

This method randomly permutes each predictor variable at a time and computes the associated reduction in predictive performance. This is similar to the variable importance measures uses for random forests, but this method currently computes using the entire training dataset, and sums the importance over each boosting iteration.

Conditional inference random forest (CIRF)

CIRF is an ensemble of individual decision trees that uses statistical theory based on covariate selection scheme during the training process. CIRF can avoids potential bias and enables the more appropriate calculation of feature importance.

R packages

The following R packages for machine learning approaches were used: *glmnet, rms, ranger, rpart, caret, xgboost, gbm, kernlab, plyr, randomForest, party, ada, naivebayes,* and *e1071.*

Table S1. Bleeding severity stratified by universal definition of perioperative bleeding in adult cardiac surgery

Bleed severity	Sternal closure delay	Blood loss within 12 h (mL)	RBC (U)	FFP (U)	Platelets (U)	Cryoprecipitate	PPC	rFVIIa	Re-exploration or tamponade
Class 0	No	<600	0	0	0	No	No	No	No
Class 1	No	601-800	1	0	0	No	No	No	No
Class 2	No	801-1000	2-4	2-4	Yes	Yes	Yes	No	No
Class 3	Yes	1001-2000	5-10	5-10	NA	NA	NA	No	Yes
Class 4	NA	>2000	>10	>10	NA	NA	NA	Yes	NA

RBC, Red blood cells; FFP, fresh frozen plasma, PCC, prothrombin complex concentrate; rFVIIa, recombined activated factor VII; NA, not applicable.

Table S2. Missing values and dispositions.

Variables	No. of missing values
NT-proBNP, (pg/ml)	66 (6.3%)
Calcium, (mmol/L)	8 (0.7%)
PT	9 (0.8%)
INR	11 (1.0%)
WBC	3 (0.2%)
HSCRP, (mg/L)	10 (0.9%)

NT-proBNP, N-terminal prohormone of brain natriuretic peptide; HSCRP, high sensitivity C reactive protein; PT, prothrombin time; INR, international normalized ratio; WBC, white blood cell

Table S3 Baseline characteristics between the training and test set

Parameter	Total (n=1045)	Training (n=732)	Test (n=313)	*P*
Age (years)	62(55-66)	62(55-67)	61(55-66)	0.801
Male, n (%)	819 (78.37)	559 (76.37)	260 (83.07)	0.017
Height (m)	1.69 (1.63-1.72)	1.68(1.62-1.72)	1.70(1.64-1.72)	0.37
Weight (kg)	72.00(65.00-80.00)	72.00(65.00-80.00)	73.00(65.00-80.00)	0.749
BSA (m^2)	1.84±0.17	1.83±0.17	1.84±0.16	0.577
Smoking history, n (%)	555 (53.11)	375 (51.23)	180 (57.51)	0.068
Angina, n (%)	988 (94.55)	699 (95.49)	289 (92.33)	0.052
Myocardial infarction, n (%)	86 (8.23)	62 (8.47)	24 (7.67)	0.714
Arrhythmia, n (%)	28 (2.68)	22 (3.01)	6 (1.92)	0.405
Previous surgery, n (%)	247 (23.64)	176 (24.04)	71 (22.68)	0.691
Diabetes, n (%)	381 (36.46)	272 (37.16)	109 (34.82)	0.484
Hyperlipidemia, n (%)	854 (81.72)	603 (82.38)	251 (80.19)	0.432
Hypertension, n (%)	650 (62.20)	459 (62.70)	191 (61.02)	0.626
Kidney failure, n (%)	35 (3.35)	21 (2.87)	14 (4.47)	0.192
Dialysis, n (%)	27 (2.58)	17 (2.32)	10 (3.19)	0.402
Chronic pulmonary disease, n (%)	17 (1.63)	11 (1.50)	6 (1.92)	0.602
Congestive heart failure, n (%)	1032 (98.76)	722 (98.63)	310 (99.04)	0.765
Anemia, n (%)	267 (25.55)	183 (25.00)	84 (26.84)	0.536
Peripheral vascular disease, n (%)	251 (24.02)	174 (23.77)	77 (24.60)	0.813
Venous disease, n (%)	62 (5.93)	46 (6.28)	16 (5.11)	0.568
Cerebrovascular disease, n (%)	113 (10.81)	84 (11.48)	29 (9.27)	0.328
Previous PTCA, n (%)	25 (2.39)	16 (2.19)	9 (2.88)	0.511
Previous thrombolysis, n (%)	6 (0.57)	5 (0.68)	1 (0.32)	0.675
CHD family history, n (%)	132 (12.63)	77 (10.52)	55 (17.57)	0.002
Preoperative statin use, n (%)	610 (58.37)	425 (58.06)	185 (59.11)	0.784
Preoperative anticoagulant use, n (%)	917 (87.75)	639 (87.30)	278 (88.82)	0.537
Antiplatelet drugs pause <5 days, n (%)	15 (1.44)	12 (1.64)	3 (0.96)	0.572
Left main coronary artery disease, n (%)	198 (18.95)	134 (18.31)	64 (20.45)	0.438
RBC, ($x10^{12}/L$)	4.45±0.52	4.44±0.52	4.45±0.54	0.778
WBC, ($x10^9/L$)	6.43(5.34-7.53)	6.36(5.31-7.41)	6.62(5.45-7.91)	0.029
PLT, ($x10^9/L$)	209(177-247)	208 (176-247)	212.00(181-246)	0.276
Platelet distribution width, (fL)	12.30(11.10-13.80)	12.30(11.20-13.80)	12.40(11.10-13.70)	0.993
Platelet volume, (fL)	10.60(10.00-11.20)	10.50(10.00-11.20)	10.60(9.90-11.20)	0.861
Platelet-large cell ration, (%)	29.50(24.60-34.70)	29.40(24.70-34.90)	29.80(24.20-34.20)	0.979
Thrombocytocrit, (%)	0.22(0.19-0.26)	0.22(0.19-0.26)	0.23(0.19-0.26)	0.286
Hemoglobin, (g/L)	137(126-146)	137 (126-146)	137 (126-147)	0.757
Total protein, (g/L)	66.10(62.90-70.20)	66.05(62.90-70.10)	66.30(62.80-70.50)	0.884
Albumin, (g/L)	40.90(38.70-43.50)	40.80(38.70-43.42)	41.10(38.70-43.60)	0.961
Potassium, (mmol/L)	4.03(3.79-4.25)	4.03(3.77-4.25)	4.03(3.82-4.26)	0.283
Sodium, (mmol/L)	141.07(139.06-142.85)	141.10(139.12-143.01)	141.00(139.00-142.50)	0.157
Calcium, (mmol/L)	2.26(2.19-2.34)	2.26(2.19-2.34)	2.26(2.18-2.33)	0.67
Glucose, (mmol/L)	5.34(4.70-6.56)	5.34(4.69-6.54)	5.30(4.72-6.63)	0.941
BUN, (mmol/L)	5.24(4.22-6.43)	5.23(4.19-6.44)	5.24(4.35-6.38)	0.949
Creatine, (μmol/L)	82.80(71.16-94.00)	82.00(70.48-94.00)	83.87(73.17-94.00)	0.288
GFR, ($ml/min/1.73m^2$)	84.28(71.53-93.92)	84.29(71.51-93.94)	84.23(72.18-93.44)	0.852
HSCRP, (mg/L)	1.34(0.65-2.97)	1.38(0.66-3.10)	1.25(0.64-2.68)	0.262

NT-proBNP, (pg/ml)	155.60(63.50-367.10)	156.35(62.98-369.52)	153.00(64.70-355.70)	0.934
PT, (s)	13.10(12.70-13.60)	13.10(12.60-13.60)	13.10(12.70-13.50)	0.657
INR, (R)	1.00(0.96-1.04)	1.00(0.96-1.04)	1.00(0.96-1.04)	0.722
CPB or not, n (%)	683 (65.36)	477 (65.16)	206 (65.81)	0.887
Operation time, (h)	3.90(3.30-4.40)	3.80(3.30-4.50)	3.90(3.40-4.40)	0.506
Blood loss, (ml)	590.06 (71.32)	589.54 (75.17)	591.30 (61.45)	0.715
Intraoperative transfusion, n (%)	26 (2.49)	14 (1.91)	12 (3.83)	0.082
Intraoperative urine output, (ml/kg/h)	3.12 (1.93)	3.16 (2.02)	3.03 (1.71)	0.329
Hemoglobin decrease, (g/L)*	25.00(16.00-33.00)	25.00(16.00-33.00)	24.00(17.00-34.00)	0.526
Postoperative first Creatine, (μmol/L)	70.64(61.29-82.19)	70.27(60.88-81.90)	71.48(62.34-83.07)	0.393
Postoperative first NT-proBNP, (pg/ml)	601.60 (352.00-1033.00)	602.10 (350.15-1060.75)	597.70 (357.30-1005.00)	0.932
Preoperative hospital LOS, (d)	6.00(4.00-9.00)	6.00(4.00-9.00)	6.00(4.00-9.00)	0.429
TRUST score	2(1-3)	2(1-3)	2(1-2)	0.413
WILL-BLEED score	1(1-4)	1(1-3)	1(1-4)	0.660
Major Bleeding, n (%)	74(7.08)	52(7.10)	22(7.01)	0.965

BSA, body surface area; PTCA, Percutaneous Transluminal Coronary Angioplasty; CHD, coronary heart disease; RBC, red blood cell; WBC, white blood cell; PLT, platelet; BUN, blood urea nitrogen; GFR, glomerular filtration rate; HSCRP, high sensitivity C reactive protein; NT-proBNP, N-terminal prohormone of brain natriuretic peptide; PT, prothrombin time; INR, international normalized ratio; LOS, length of stay.

*The difference between the preoperative last hemoglobin and the first hemoglobin after surgery.

Table S4. Variables included in statistical and machine learning models

Parameter	Logistic regression	ML algorithms*
Age (years)		√
Male, n (%)		√
Height (m)		√
Weight (kg)		√
BSA (m^2)	√	√
Smoking history, n (%)		√
Angina, n (%)		√
Myocardial infarction, n (%)		√
Arrhythmia, n (%)		√
Previous surgery, n (%)		√
Diabetes, n (%)	√	√
Hyperlipidemia, n (%)		√
Hypertension, n (%)	√	√
Kidney failure, n (%)		√
Dialysis, n (%)		√
Chronic pulmonary disease, n (%)		√
Congestive heart failure, n (%)		√
Anemia, n (%)		√
Peripheral vascular disease, n (%)		√
Venous disease, n (%)		√
Cerebrovascular disease, n (%)		√
Previous PTCA, n (%)		√
Previous thrombolysis, n (%)		√
CHD family history, n (%)		√
Preoperative statin use, n (%)		√
Preoperative anticoagulant use, n (%)		√
Antiplatelet drugs pause <5 days, n (%)		√
Left main coronary artery disease, n (%)		√
RBC, ($x10^{12}/L$)		√
WBC, ($x10^9/L$)		√
PLT, ($x10^9/L$)		√
Platelet distribution width, (fL)		√
Platelet volume, (fL)		√
Platelet-large cell ration, (%)		√
Thrombocytocrit, (%)		√
Hemoglobin, (g/L)		√
Total protein, (g/L)	√	√
Albumin, (g/L)		√
Potassium, (mmol/L)		√
Sodium, (mmol/L)		√
Calcium, (mmol/L)		√
Glucose, (mmol/L)		√
BUN, (mmol/L)		√
Creatine, (μmol/L)	√	√
GFR, ($ml/min/1.73m^2$)		√
HSCRP, (mg/L)		√
NT-proBNP, (pg/ml)	√	√
PT, (s)		√

INR, (R)		√
CPB or not, n (%)		√
Operation time, (h)		√
Blood loss, (ml)	√	√
Intraoperative transfusion, n (%)	√	√
Intraoperative urine output, (ml/kg/h)		√
Hemoglobin decrease, (g/L)		√
Postoperative first Creatine, (μmol/L)		√
Postoperative first NT-proBNP, (pg/ml)		√
Preoperative hospital LOS, (d)		√
TRUST score		√
WILL-BLEED score		√

*Support vector machines, stochastic gradient boosting, extreme gradient boosting, random forest, conditional inference random forest, boosted classification trees, Naïve Bayes and bagged CART.

Table S5. Abbreviations

ML = Machine learning
CABG = Coronary artery bypass graft
UDPB = Universal definition of perioperative bleeding
AUC = Area under the receiver operating characteristic curves
TRUST = Transfusion risk understanding scoring tool
LR = Logistic regression
AIC = Akaike information criterion
SVM = Support vector machines
SGBT = Stochastic gradient bossting
XGBoost = Extreme gradient boosting
RF = Random forest
CIRF = Conditional inference random forest
NB = Naïve Bayes
CART = Classification and regression tree
ROC = Receiver-operating-characteristic curve
BSA = Body surface area
STS PROM = Society of Thoracic Surgeons Predicted
